# Expression of circulating angiotensin‐converting enzyme 2 in children with asthma and the effects of inhaled corticosteroids

**DOI:** 10.1002/iid3.922

**Published:** 2023-07-27

**Authors:** Xiang Ma, Lesen Liu, Jing Sun, Yun Zhang, Yanqin Liu, Miao Liu, Bing Wang, Yuna Chang, Yuling Han, Zhongtao Gai

**Affiliations:** ^1^ Department of Respiratory Diseases Children's Hospital affiliated to Shandong University Jinan China; ^2^ Jinan Key Lab of Respiratory Diseases for Children Jinan Children's Hospital Jinan China; ^3^ Department of Infectious Diseases Huaiyin People's Hospital Jinan China

**Keywords:** angiotensin‐converting enzyme 2, asthma, children, COVID‐19, inhaled corticosteroids

## Abstract

**Background:**

The global spread of coronavirus disease 2019 (COVID‐19) has resulted in a significant disease burden, yet asthma patients do not have the expected high morbidity and mortality rates in the pandemics of COVID‐19.

**Objective:**

To find the difference of angiotensin‐converting enzyme 2 (ACE2) in asthma and nonasthma children and evaluate the effect of inhaled corticosteroids (ICS) on its expression.

**Methods:**

The ACE2, immunoglobulin E (IgE), and eosinophils were tested in different children.

**Results:**

A total of 157 children aged 3–16 years were enrolled. The expression of ACE2 in asthma children were lower than nonasthma children (*T* = −2.512, *p* = .013). Allergic nonasthma children had a significant higher ACE2 expression than children with allergic asthma (*p* = .013) and nonallergic asthma (*p* = .029). The expression of ACE2 had no significant difference between first‐diagnosed asthma children and that had been treated with ICS for ≥6 months (*F* = 0.028, *p* = .598). The allergic asthma children showed a significantly higher eosinophils cells (EC) count than the allergic nonasthma (*W* = 200, *p* < .001) and nonallergic nonasthma children (*W* = 1089, *p* < .001). Nonallergic asthma children also had a significant higher EC count than the allergic non‐asthma (*W* = 182.5, *p* < .001) and nonallergic non‐asthma (*W* = 200.5, *p* < .001) children. There was no significant difference in IgE levels between asthmatic children and non‐asthmatic children (*W* = 2792.5, *p* = .18).

**Conclusion:**

Circulating ACE2 levels in asthmatic children were lower than those in non‐asthmatic children and ICS treatment for ≥6 months did not affect the expression of ACE2 in peripheral blood in the asthma children.

## Key Points

In the present study, we analyze the expression of circulating angiotensin‐converting enzyme 2 in asthmatic children and the effects of inhaled corticosteroids. And we found that the children with asthma had lower ACE2 level than no‐asthmatic children. Furthermore, there were no significant difference between the asthmatic children with or without ICS treatment. It's very helpful to get further understanding about the relationship between asthma and COVID‐19 in children.

## 1 INTRODUCTION

Angiotensin‐converting enzyme 2 (ACE2) is a membrane protein confirmed as the main host cell receptor of SARS‐CoV‐2. ACE2 plays a crucial role in the entry of this virus into cells, thereby eventually causing the infection.^1^ A high ACE2 expression corresponds with increased susceptibility to SARS‐CoV in vitro^2^ and may be related to clinical prognosis.^3^ Substantial clinical data have shown that this new virus van infect all organs expressing ACE2 such as the lungs, digestive system organs, kidney, heart, and even the reproductive system organs.^4^, ^5^, ^6^ In other words, basic and clinical data suggested that the ACE2 expression is closely related to the clinical manifestations and severity of COVID‐19.^7^, ^8^

Bronchial asthma is a chronic airway inflammatory disease and a common chronic underlying disease. Numerous pathogenic infections such as respiratory syncytial virus, rhinovirus, and *Mycoplasma pneumoniae* can induce the occurrence of or worsen asthma.^9^, ^10^, ^11^ During the COVID‐19 pandemic, asthma patients reportedly have higher infection risks and more severe symptoms than healthy people. However, existing clinical data indicate various behaviors and outcomes in different countries. Data from China have shown that asthma patients account for only 0%–0.9% in the reported cases,^12^, ^13^, ^14^, ^15^ but the CDC has reported 18.8% of children with asthma.^16^

Interestingly, substantial variations exist within the same disease status, particularly ACE2 expression in asthmatics and the effects of inhaled corticosteroids (ICS) on ACE2. These variations may be explained by issues such as race and data bias, as well as the relationship between the disease itself or the treatment drug and the virus infection.^17^, ^18^, ^19^, ^20^ Accordingly, the present experimental study was designed to understand the differences in ACE2 expression in asthmatic and healthy children and the effects of ICS on changes in ACE2 levels.

## 2 MATERIALS AND METHODS

### 2.1 Study design and population

A prospective controlled study involving asthma and healthy children among outpatients and inpatients in Jinan Children's Hospital was conducted from March 1 to June 15, 2020. All children were 3–16 years old and were of Han nationality from Shandong Province, China. The diagnostic criteria for asthma and treatment of ICS were in accordance with the standards of the Global Initiative for Asthma (GINA)^21^ and Chinese Children's Asthma Prevention and Treatment Guidelines.^22^ We excluded all patients with immunodeficiency, tuberculosis infection, fungal infection, and congenital heart and lung diseases. Patients infected with COVID‐19, diabetic patients, and patients treated with ACE inhibitors such as captopril, enalapril, and lisinopril were not included in the study. All children had a family income of 200,000–280,000 Yuan per year, and their body mass index was between 19 and 24.

### 2.2 Ethics

This study was conducted in accordance with the Declaration of Helsinki. Researchers obtained written consent from parents and guardians of all patients. The ethics committee reference number was ETYY‐2020237 in the Children's Hospital affiliated to Shandong University.

### 2.3 Patient recruitment and group design

All patients were divided into four groups (allergic asthma, nonallergic asthma, allergic nonasthma, and nonallergic nonasthma) according to whether they had asthma or not and had atopic constitution (i.e., had symptoms of at least one allergic condition, such as allergic rhinitis, atopic dermatitis, food allergy, and allergic conjunctivitis and/or positive skin test). Nonallergic nonasthma children were healthy and had no atopic disease history.

All asthma children were divided into two subgroups. Subgroup 1 was the asthma initial diagnosis group. Children in this subgroup were diagnosed with asthma for the first time and had not been treated with any asthma medication for the last 3 months. Subgroup 2 was the asthma treatment group with ICS. Children in this subgroup had received regular (daily) ICS for at least 6 months (the daily dose of ICS was between 200 and 500 µg equivalent to budesonide).

### 2.4 Collection of blood specimens

Eosinophil count (EC) was determined using whole blood. Serum total ACE2 (item number: CSB‐E04489h; CUSABIO) and IgE (item number: CSB‐E04980h; CUSABIO) were quantitated using enzyme‐linked immunosorbent assay. The limit of detection of ACE2 and IgE were 0.059 pg/mL and 0.172 ng/mL, respectively. A serum‐separator tube was used, and samples were allowed to clot for 2 h at room temperature or overnight at 4°C before centrifugation for 15 min at 1000*g*. The serum was removed and assayed immediately or separately packed, and samples were stored at −20°C or −80°C.

### 2.5 Statistical analysis

For continuous data compared between two independent groups, *p* values were derived by one‐way Student's *t*‐test to determine differences between groups with normally distributed data. Wilcoxon rank sum test was used with other data. For continuous data compared between multiple groups, *p* values were derived by one‐way analysis of variance to determine differences between groups with normally distributed data. Kruskal–Wallis *H* test was used with other data. Variations in statistical significance were further subjected to post hoc pairwise analysis by applying the Wilcoxon test and Bonferroni's correction. Two‐tailed *p* < .05 were considered statistically significant. Statistical analyses were performed using R statistical software (version 4.1.2).

## 3 RESULTS

### 3.1 General information

A total of 157 children (77 asthma and 80 nonasthma) were included in this study (Table 1). Among 77 children with asthma, 39 were first diagnosed asthma (without ICS treatment), and 38 received regular daily ICS treatment for more than 6 months.

**Table 1:** General information on the study.

Subgroup	*N*	Male (%)	Age (year)
Allergic asthma	45	33 (73.3)	7.03 (3.5–12.4)
Nonallergic asthma	32	19 (59.4)	7.20 (3.1–13.8)
Nonallergic nonasthma	35	21 (60.0)	5.02 (3–10)
Allergic nonasthma	45	25 (55.6)	7.26 (3–14)

### 3.2 Expression of ACE2 in different groups

**Figure 1:**
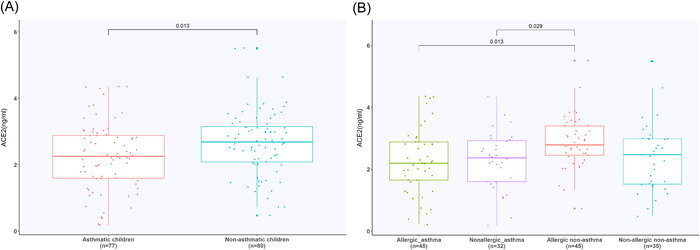
Expression of ACE2 in different groups. (A) Expression of ACE2 in asthma and nonasthma children. (B) Expression of ACE2 in four subgroups. ACE2, angiotensin‐converting enzyme 2.

ACE2 expression in circulating blood in asthma children was significantly lower than that in nonasthma children (*T* = −2.512, *p* = .013) (Figure 1A). Significant differences were also observed among the four subgroups (*F* = 3.766, *p* = .012). Allergic nonasthma children had a significantly higher level than children with allergic and nonallergic asthma (*p* = .013 and .029, respectively). No significant difference existed between the other subgroups (Figure 1B).

### 3.3 Expressions of EC and IgE in different groups

**Figure 2:**
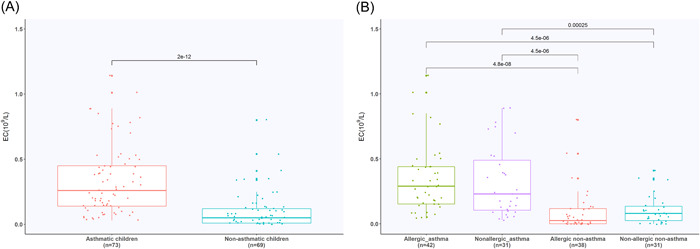
Count of EC in different groups. (A) Count of EC in asthma and nonasthma children. (B) Count of EC in four subgroups. EC, eosinophil count.

**Figure 3:**
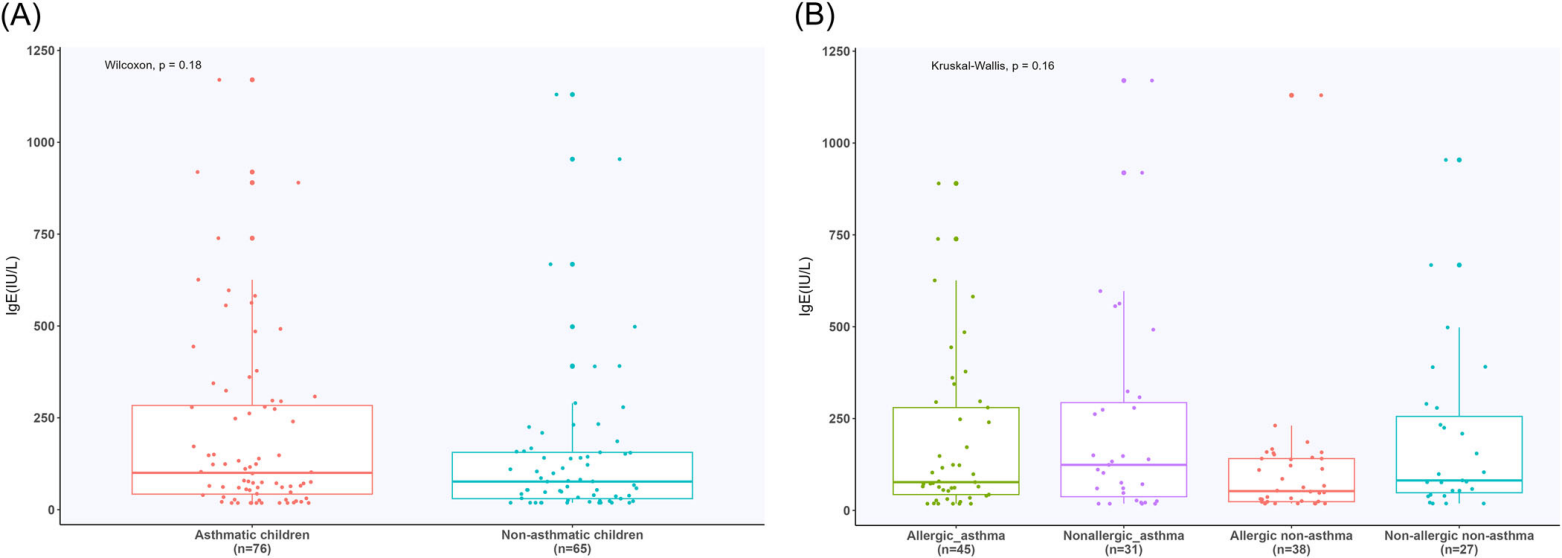
IgE in different groups. (A) IgE in peripheral blood in asthma and nonasthma children. (B) IgE in peripheral blood in four subgroups. IgE, immunoglobulin E.

A significant difference was found in the EC count between the asthma and nonasthma groups (*W* = 1396, *p* < .01). When the four subgroups were compared, the allergic asthma children showed a significantly higher EC count than allergic nonasthma (*W* = 200, *p* < .001) and nonallergic nonasthma (*W* = 1089, *p* < .001) children. Nonallergic asthma children had a more significant higher EC account than the allergic nonasthma (*W* = 182.5, *p* < .001) and nonallergic nonasthma (*W* = 200.5, *p* < .001) children (Figure 2). No significant differences existed in IgE levels between asthma and nonasthma (*W* = 2792.5, *p* = .18) children, nor between the four subgroups (*χ* ^2^ = 5.2041, *p* = .16) (Figure 3).

### 3.4 Effect of ICS on ACE2 expression

Thirty‐nine children with asthma were initially diagnosed asthma and did not accept any ICS, and 38 children with asthma had received standardized ICS treatment every day for more than 6 months. No significant difference existed in ACE2 expression among these asthmatic children (*F* = 0.028, *p* = .598).

## 4 DISCUSSION

As a chronic lung disease, asthma is considered to be a clinical problem affected by COVID‐19. However, based on some current data, asthma patients do not show a high prevalence in the COVID‐19 epidemic.^7^, ^8^, ^9^, ^10^ ACE2 level is considered as one of the most important factors affecting COVID‐19 infection. Accordingly, we investigated in the current work whether ACE2 expression in asthmatic patients was lower than that in other populations and whether ICS treatment affected ACE2 expression. We found that ACE2 expression in asthma children was lower than that in nonasthma children. This result was theoretically consistent with the low incidence of asthma in COVID‐19 infection. However, such an outcome differed from the results of numerous studies, and some are even completely consistent. For example, one study reported no difference in the expression of ACE2 genes in the sputum of 330 asthmatic and 79 healthy participants. Among the asthmatic population, a high expression of ACE2 is reportedly associated with males, African Americans, and those with history of diabetes mellitus.^23^ Peter et al. also reported that in bronchial biopsies, no differences are observed in ACE2 gene expressions between healthy volunteers and patients with asthma, regardless of whether the asthma is mild, moderate, or severe.^24^ Conversely, another study has found that moderate and high allergic sensitizations among children with asthma are associated with progressively more substantial reductions in ACE2 compared with children having asthma but having no/minimal allergic sensitization.^25^ Some studies even have opposite results or findings. Public data sets show that ACE2 is upregulated in the blood for the asthmatic population.^26^ Radzikowska et al. found high expression levels of ACE2‐ and CD147‐related genes in bronchial biopsy, BAL, or blood in asthmatic patients.^4^ Ortiz et al. observed increased ACE2 only in children below 10 years old and in the bronchioles of individuals without chronic comorbidities.^27^ In summary, ACE2 expression may be related to the different test populations, specimen sources, experimental methods, ethnicities, ages, regions, and other unknown factors. Therefore, the relationship between ACE2 expression in the human body and pathogenesis of asthma should be further analyzed.

ICS, alone or combined with bronchodilators (e.g., β2 receptor agonists) is used extensively to treat asthma (GINA 2020) and undoubtedly reduces the rate of asthma exacerbation.^21^ Taking ICS can reportedly be beneficial in dealing with coronavirus infections. Peters et al.^20^ found that ICS significantly decreases ACE2 expression in the sputum of asthma patients. Yamaya et al.^28^ reported that pretreatment of human respiratory epithelial cells with budesonide, combined with glycopyrronium and formoterol in vitro may inhibit coronavirus HCoV‐229E replication and cytokine production. An in vitro study has suggested that ciclesonide blocks SARS‐CoV‐2 RNA replication^29^ and inhibits SARS‐CoV‐2 cytopathic activity.^30^ ICS use in patients at risk of acute respiratory distress syndrome (ARDS) has been shown to improve physiology and reduce levels of inflammatory markers.^31^ A 50%‐reduction in ARDS has been observed in at‐risk patients using ICS before admission to hospital, even after controlling for age, sex, and chronic respiratory diseases.^32^ However, in vitro studies have shown that corticosteroids possibly impair antiviral innate immune responses,^33^, ^34^ thereby resulting in delayed virus clearance.^35^ The current study failed to find significant difference in the blood levels of ACE2 between untreated asthmatic children and asthmatic children treated with ICS. The possible reasons for these differences are as follows. First, the sources of this study differed because the ACE2 expression levels in different tissues were inconsistent. For example, the majority of related studies have shown that it is highly expressed primarily in epithelial cells in the lungs.^3^, ^36^, ^37^ Second, the test subjects differed. Some experiments have used animal models, whereas some have been conducted among humans. A few have been performed on adults, whereas some others have focused on children. Third, the ICS doses varied. The majority of related studies have not compared the effects of the different therapeutic doses of ICS. Evidently, the therapeutic doses of ICS are bound to change owing to the different treatment levels of asthma. Fourth, other reasons may be cited. For example, the severity of asthma, duration of treatment time, observation time of the study, and detection methods may also lead to different results. Hence, although observations for ICS and COVID‐19 do not provide a complete scenario, they should not be disregarded because patients are expected to have pre‐existing, serious lung conditions.

The current work included 157 children, which was extremely challenging and difficult due to the low prevalence of pediatric asthma. It had the approval of an ethics committee and followed the rules set out in the Declaration of Helsinki. However, this study had some limitations. First, important variables that may exert an effect on results were not included. These variables include medication adherence, inhaler techniques, lung function, the severity of asthma, and controlled status. Second, probable associations between variables and outcome were not evaluated.

During the COVID‐19 pandemic, the relationship between asthma and COVID‐19 is unclear. As a therapeutic intervention, ICS should be continuously studied, and clinical trials must assess its efficacy in COVID‐19. Accordingly, further in‐depth clinical and basic research is imperative.

## AUTHOR CONTRIBUTIONS

**Xiang Ma**: Conceptualization; writing—review and editing. **Lesen Liu**: Data curation. **Jing Sun**: Investigation; resources. **Yun Zhang**: Investigation; resources. **Yanqin Liu**: Data curation; software. **Miao Liu**: Methodology; supervision. **Bing Wang**: Software; validation. **Yuna Chang**: Supervision; visualization. **Yuling Han**: Project administration; visualization. **Zhongtao Gai**: Conceptualization; writing—original draft.

## CONFLICT OF INTEREST STATEMENT

The authors declare no conflict of interest.

## Data Availability

The authors have nothing to report.
